# Standardizing haematopoietic cell transplants in China

**DOI:** 10.1186/s13045-018-0565-9

**Published:** 2018-03-02

**Authors:** Robert Peter Gale

**Affiliations:** Haematology Research Centre, Division of Experimental Medicine, Department of Medicine, Imperial College London, London, SW7 2AZ UK

*You cannot open a book without learning something*. (没有学到东西就无法打开书)

*Confucius*

This *expert consensus* statement is an *opus magnum* written by 19 Chinese leaders in blood disorders, especially in hematopoietic cell transplants. The statement covers almost every category of neoplastic and non-neoplastic conditions and many aspects of transplants including appropriate recipients, donors, pretransplant conditioning regimens, graft types and graft-versus-host disease (G*v*HD) prevention. It also gives us a snapshot of what is going on in transplants today in China.

First, why should we be interested in what is going on in China? For many reasons, China is one of the world’s oldest civilizations and the most populous nation. One in every five humans is Chinese. Prominence of China on the world stage is growing rapidly including in science and medicine. And our Chinese colleagues have done more HLA-haplotype-matched transplants than everyone else combined, so there is much to be learned from their experience. As the authors indicate, almost 20% of all transplants worldwide are being done in China including 40% of HLA-haplotype-matched transplants. Wow.

It would be impossible for me to comment on the entire content of this document; it might also take a lifetime. Instead my focus is on some key concepts and illustrate these with examples. First, the typescript is an expert consensus statement. As such, we might ask ourselves what exactly these words mean. There are many definitions of *consensus*. For example, the Oxford English Dictionary defines consensus as *a general agreement* derived from the Latin *consens*, to agree. Note that there is no implication a consensus is correct. For example, there was consensus amongst Western religious leaders in the Middle Ages that the world was flat, an idea already known to be wrong in the Western Han Dynasty (西漢). Also, before Copernicus and Galileo, there was Western consensus that the Earth was the centre of the Universe, a notion disproved by Chinese astronomers in the Shang Dynasty (商; Fig. 1).

**Fig. 1:**
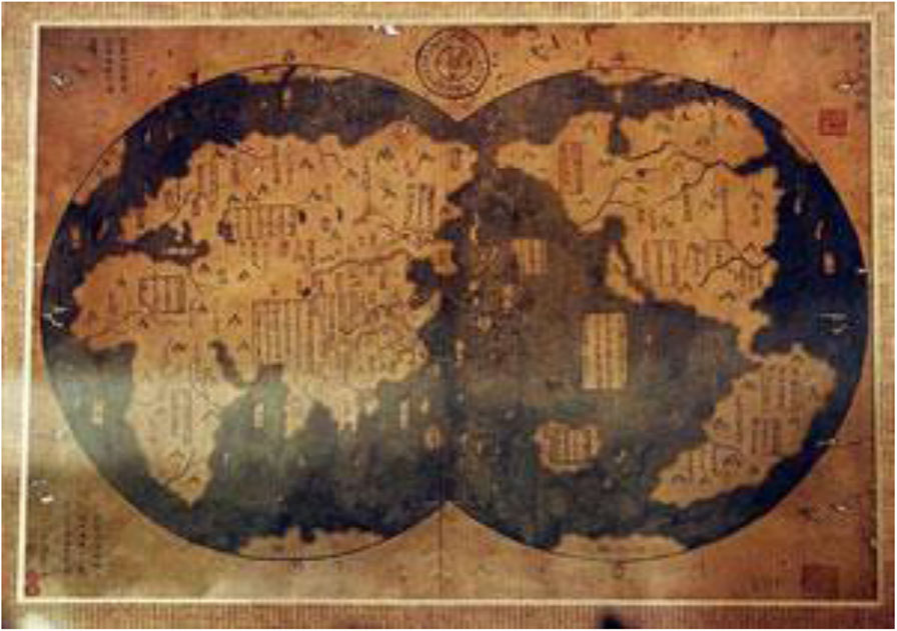
1418 Ming Dynasty (明) map

The most important variables in analyzing the validity of consensus statements and guidelines is how they were developed and the quality of evidence on which they are based. The development process can range from friends or colleagues around a table over drinks late one evening to a highly structured, evidence-based reiterative process such as the RAND-Delhi expert consensus panel process [1, 2], more on the quality of evidence below.

Although c*onsensus statements* and *clinical practice guidelines* are intended to provide guidance to clinicians, they differ. A consensus statement is developed by an independent panel of experts, usually multi-disciplinary, convened to review the research literature in an evidence-based manner to advance understanding of an issue. In contrast, a clinical practice guideline produces statements informed by a systematic review of the evidence. Although consensus statements address topics in which the evidence base is less extensive compared to clinical practice guidelines, their development should still be methodologically rigorous and transparent. Organizations such as Appraisal of Guidelines for Research and Evaluation [3], Institute of Medicine (now National Academy of Medicine) [4], Guidelines International Network [5] and Oxford Univ. Centre for Evidence-Based Medicine developed [6] criteria to ensure objective, scientifically valid and consistent standards for development and reporting of high-quality guidance documents.

In trying to analyze the typescript by Huang and colleagues, we are hampered by the lack of details on the consensus process used. Hopefully, the authors will publish these in a supplement. As for the content, there are too many recommendations to discuss each in detail. However, there are several recurrent themes which have perplexed transplant experts for several decades: (1) who should receive a transplant, (2) when and (3) how? Correct answers to these questions require randomized clinical trials which account for all possible participants followed prospectively. Unfortunately, these are rarely available. Mostly what we have are data from small phase 2 studies and or large observational databases. Elsewhere, my colleagues and I discuss limitations, mostly selection biases, of trying to deduce the *truth* from such datasets [7].

Expert consensus statements and clinical practice guidelines are part of a movement termed *evidence-based medicine* (the implication being everything done before 1997 was voodoo). Proponents of this approach claim medical practice should be data-driven. Perfectly logical. However, on closer examination, the quality of evidence on which medical decisions are based is often poor. In several surveys of clinical studies published in high-quality medical journals, about one half of interventions were subsequently shown to be either unproved, ineffective or harmful [8–10]. Such changes, referred to as *medical reversal*, are reviewed elsewhere [11]. Widespread use of high-dose chemotherapy and autotransplants in women with high-risk breast cancer is a relevant example of a medical reversal [12, 13]. Other medical reversals have far greater consequences. Some examples are as follows: It now seems there is no benefit of giving statins to healthy persons 40–75 years old with no history of cardio-vascular disease, no risk factors and a projected 10-year risk of heart disease < 7.5% [14]. This is most people in China, the USA and EU who are currently taking statins. It also seems that after 40 years with 500,000 procedures *per* year in the USA and EU, percutaneous coronary intervention in persons with stable angina is no better than medical therapy [15]. The bottom line is we often know much less than we think.

Not everyone believes in evidence-based medicine or thinks the movement has gone too far. These people argue medicine is more of an *art* than a *science*, and limiting medical practice to expert consensus statements and clinical practice guidelines removes focus from the individual patient. This position also has major limitations and ignores much of the albeit imperfect data available to assist in clinical decision-making. Perhaps both viewpoints are correct [16].

The authors say their consensus document complements the National Comprehensive Cancer Centre (NCCN) and British Committee for Standard in Haematology (BCSH) clinical practice guidelines. Let us look at two recommendations from the Huang typescript, 1 controversial and 1 not. In persons with acute myeloid leukaemia (AML) *< 60 years with intermediate-risk disease according to the WHO risk stratification*, the authors recommend an allotransplant. The 2017 NCCN AML clinical practice guideline, presumably looking at the same evidence, suggests several therapies as being comparable: (1) a clinical trial, (2) an allotransplant from a sibling or alternative donor (a HLA-haplotype-matched donor is not mentioned but is probably reasonable, especially in China), or (3) high-dose cytarabine [17] whereas the European LeukemiaNet (ELN) recommendation states: *Allogeneic HCT is generally recommended when the relapse incidence without the procedure is expected to be > 35 to 40%* [18]. How do we reconcile these discordant recommendations? We cannot. But both cannot be correct.

But if we look at recommendations for *persons < 60 with Ph1-chromsome-positive acute lymphoblastic leukaemia (ALL)*, recommendations for a transplant from Huang and co-workers and from the NCCN are concordant as are recommendations from several other groups [16]. This is consensus but, as discussed above, consensus does not guarantee truth. In statistics, this is *precision* (getting the same answer each time) but not *accuracy* (getting the correct answer at least some of the time). For example, some recent data suggest comparable outcomes with second-generation tyrosine kinase-inhibitors (TKIs) and allotransplants (reviewed in reference [19]). Again, this question can only be resolved in a randomized clinical trial, unlikely to be done. What we are striving for is precision and accuracy, a laudable goal but one difficult or impossible to achieve in medicine.

These concordances, discordances and uncertainties raise the important issue of how to compare levels of evidence, levels of recommendations and applicability of recommendations to general practice. An extensive review of this topic is beyond this editorial, but I summarize these topics in Tables 1, 2 and 3. Not all evidence is of comparable quality, and experts should identify the quality of evidence underlying their consensus statements and strength of their recommendations. The validity of recommendations is a separate issue which should be evaluated by persons external to the authors using the Grading of Recommendations Assessment, Development and Evaluation (GRADE) approach, a method of assessing the certainty of evidence (also known as quality of evidence or confidence in effect estimates) and the strength of recommendations in health care [20].

**Table 1:** Scale of quality of evidence for a therapy recommendation

1a:	Systematic reviews (with homogeneity) of randomized controlled trials
1b:	Individual randomized controlled trials (with narrow confidence interval)
1c:	All or none randomized controlled trials
2a:	Systematic reviews (with homogeneity) of cohort studies
2b:	Individual cohort study or low quality randomized controlled trials (e.g. < 80% follow-up)
2c:	“Outcomes” research; ecological studies
3a:	Systematic review (with homogeneity) of case-control studies
3b:	Individual case-control study
4:	Case-series (and poor quality cohort and case-control studies)
5:	Expert opinion without explicit critical appraisal or based on physiology, bench research or “first principles”

**Table 2:** Grades of recommendation

A	At least one meta-analysis, systematic review or randomized controlled trial (RCT) rated as 1++, and directly applicable to the target population; or a systematic review of RCTs or a body of evidence consisting principally of studies rated as 1+, directly applicable to the target population, and demonstrating overall consistency of results
B	A body of evidence including studies rated as 2++, directly applicable to the target population and demonstrating overall consistency of results; or extrapolated evidence from studies rated as 1++ or 1+
C	A body of evidence including studies rated as 2+, directly applicable to the target population and demonstrating overall consistency of results; or extrapolated evidence from studies rated as 2++
D	Evidence level 3 or 4; or extrapolated evidence from studies rated as 2+

**Table 3:** Grading of Recommendations Assessment, Development and Evaluation (GRADE)

Code	Quality of evidence	Definition
A	High	Further research is very unlikely to change our confidence in the estimate of effect. • Several high-quality studies with consistent results • In special cases: one large, high-quality multi-centre trial
B	Moderate	Further research is likely to have an important impact on our confidence in the estimate of effect and may change the estimate. • One high-quality study • Several studies with some limitations
C	Low	Further research is very likely to have an important impact on our confidence in the estimate of effect and is likely to change the estimate. • One or more studies with severe limitations
D	Very low	Any estimate of effect is very uncertain. • Expert opinion • No direct research evidence • One or more studies with very severe limitations

Huang and co-workers indicate their goal is to standardize transplant therapy in China. This may be admirable (see below), but they concede their consensus conclusions may not apply to other venues. This is reasonable. Consider, for example, PCR-testing for *BCRABL1* in persons with chronic myeloid leukaemia receiving TKIs where we found only occasional follow-up testing in Chinese after starting therapy [21].

Is standardization of therapy a valid goal? Yes and no. Take, for example, nuclear reactor design. In France, most nuclear reactors have the same design which works well. When a problem is detected in one reactor, a fix can be applied to the others. In contrast to this is in the USA where almost every reactor design is different. Detection of a problem at one reactor is not easily applied to potential problems at others. This seems a strong argument for standardization. However, what if France had selected an imperfect reactor design and then applied it to all its reactors? The history of the Soviet Union in the twentieth century is a good example of standardization with disastrous consequences. When the truth is not known, an argument can be made for encouraging variability.

In the past two decades, Chinese haematology has made rapid advances. Chinese transplant specialists have benefited from several global developments including (1) progress in transplant strategies; (2) new methods for clinical trial designs and statistical analyses; (3) multi-centre, national and international cooperation in clinical trials; and (4) standardization of some clinical transplant-related practices. Standardizing transplant practices in China, encouraged by this typescript, should allow Chinese investigators to develop a large (everything is large in China) observational database similar to the Centre for International Blood and Marrow Research (CIBMTR) and European Bone Marrow Transplant Group (EBMT) databases. It should also facilitate establishing a national clinical trial research organization to evaluate new therapies in phase 2 trials and to compare prior therapies such as the Chinese approach to HLA-haplotype-matched transplants with other approaches such as posttransplant cyclophosphamide. And we should not forget what China has given us in these two decades including arsenic for acute progranulocytic leukaemia and the so-called *Beijing protocol* for HLA-haplotype-matched transplants.

Guidance documents are an essential part of oncology care and should be subjected to a rigorous and validated development process. The bottom line is expert consensus statements such as that from Huang and colleagues are likely to be useful in standardizing transplant strategies in a huge, diverse country such as China for reasons I discuss above. Their recommendations are mostly sensible, especially in a China context. Which of their recommendations will withstand scrutiny in future randomized clinical trials remains to be determined. However, such trials are unlikely to be done and, if done, conclusions unlikely to be widely believed. As such, we are left with expert consensus statements and clinical practice guidelines, all seemingly evidence-based but sometimes contradictory. The challenge is for haematologists to make appropriate patient-level decisions taking into consideration potential benefits and risks. Huang and colleagues have helped Chinese haematologists to do so with this typescript.

## References

[1] https://en.wikipedia.org/wiki/Delphi_method. Accessed 2 Jan 1918.

[2] Gale RP, Park RE, Dubois R, Bitran J, Buzdar A, Hortobagyi G (2000). Delphi-consensus panel analysis of appropriateness of high-dose chemotherapy and blood cell or bone marrow autotransplants in women with breast cancer. Clin Transpl.

[3] Browers MC, Kho ME, Browman GP, Burgers JS, Cluzeau F, Feder G (2010). AGREE II: advancing guideline development, reporting and evaluation in health care. CMAJ.

[4] Graham RMM, Miller Wolman D, Greenfield S, Steinberg E, Editors. Committee on standards for developing trustworthy clinical practice guidelines. Clinical practice guidelines we can trust: Institute of Medicine (US) committee on standards for developing trustworthy clinical practice guidelines; Washington (DC): National Academies Press; 2011. 24983061

[5] Guidelines International Network. http://www.g-i-n.net/. Accessed 19 Dec 1917.

[6] http://www.cebm.net/. Accessed 31 Dec 1917.

[7] Gale RP, Eapen M, Logan B, Zhang MJ, Lazarus HM (2009). Are there roles for observational database studies and structured quantification of expert opinion to answer therapy controversies in transplants?. Bone Marrow Transplant.

[8] Prasad V, Gail V, Cifu A (2011). The frequency of medical reversal. Arch Intern Med.

[9] Ioannidis JP (2005). Contradicted and initially stronger effects in higher cited clinical research. JAMA.

[10] SPACE FOR BR MED J. http://ebm.bmj.com/. Accessed 6 Feb 2018.

[11] Prasad VK, CIfu AS. Ending medical reversal: improving outcomes, saving lives. 1st Edition. Baltimore, John Hopkins University Press; 2005.

[12] Howard DH, Kenline C, Lazarus HM, Lemaistre CF, Maziarz RT, McCarthy PL (2011). Abandonment of high-dose chemotherapy/hematopoietic cell transplants for breast cancer following negative trial results. Health Serv Res.

[13] Berry DA, Ueno NT, Johnson MM, Lei X, Caputo J, Rohenhuis S (2011). High-dose chemotherapy with autologous stem-cell support as adjuvant therapy in breast cancer: overview of 15 randomized trials. J Clin Oncol.

[14] https://www.uspreventiveservicestaskforce.org/Page/Document/RecommendationStatementFinal/statin-use-in-adults-preventive-medication1. Accessed 6 Jan 1918.

[15] Al-Lamee R, Thompson D, Sen S, Tang K, Davies J, Keeble T (2018). Percutaneous coronary intervention in stable angina (ORBITA): a double-blind, randomized controlled trial. Lancet.

[16] https://www.nytimes.com/2017/12/27/upshot/what-we-mean-when-we-say-evidence-based-medicine.html. Accessed 30 Dec 1917.

[17] https://www.nccn.org/professionals/physician_gls/pdf/aml.pdf. Accessed 30 Dec 1917.

[18] Döhner H, Estey E, Grimwade D, Amadori S, Appelbaum FS, Büchner T (2017). Diagnosis and management of AML in adults: 2017 ELN recommendations from an international expert panel. Blood.

[19] Ravandi F (2017). Current management of Philadelphia chromosome positive ALL and the role of stem cell transplantation. Hematology Am Soc Hematol Educ Program.

[20] http://www.gradeworkinggroup.org/. Accessed 31 Dec 1917.

[21] Jian Q, Gale RP (2016). Molecular monitoring of tyrosine kinase inhibitor therapy of chronic myeloid leukemia in China. J Cancer Res Clin Oncol.

